# Image-Based Machine Learning Characterizes Root Nodule in Soybean Exposed to Silicon

**DOI:** 10.3389/fpls.2020.520161

**Published:** 2020-10-28

**Authors:** Yong Suk Chung, Unseok Lee, Seong Heo, Renato Rodrigues Silva, Chae-In Na, Yoonha Kim

**Affiliations:** ^1^Department of Plant Resources and Environment, Jeju National University, Jeju-si, South Korea; ^2^Smart Farm Research Center, Korea Institute of Science and Technology, Gangneung-si, South Korea; ^3^Department of Horticulture, Kongju National University, Yesan, South Korea; ^4^Institute of Mathematics and Statistics, Federal University of Goiás, Goiânia, Brazil; ^5^Department of Agronomy, Gyeongsang National University, Jinju-si, South Korea; ^6^School of Applied Life Science, Kyungpook National University, Daegu, South Korea

**Keywords:** phenomics, root phenotype, nodule count, nodule size, legume, machine learning, image process, high-throughput phenotyping

## Abstract

Silicon promotes nodule formation in legume roots which is crucial for nitrogen fixation. However, it is very time-consuming and laborious to count the number of nodules and to measure nodule size manually, which led nodule characterization not to be study as much as other agronomical characters. Thus, the current study incorporated various techniques including machine learning to determine the number and size of root nodules and identify various root phenotypes from root images that may be associated with nodule formation with and without silicon treatment. Among those techniques, the machine learning for characterizing nodule is the first attempt, which enabled us to find high correlations among root phenotypes including root length, number of forks, and average link angles, and nodule characters such as number of nodules and nodule size with silicon treatments. The methods here could greatly accelerate further investigation such as delineating the optimal concentration of silicon for nodule formation.

## Introduction

Soybean is regarded as one of the three major crops in the world because of its nutritional value (Bellaloui et al., 2013). For this reason, soybean is broadly cultivated not only for its use as an ingredient in foods, such as soy source, soybean paste, and tofu but also for livestock feed (He and Chen, 2013). Global soybean demands have grown steadily in recent years (Armah et al., 2011), fueling the application of chemical nutrients to increase grain yield. As a result, nitrogen (N), potassium (K), and phosphorus (P) are widely used in crop cultivation (Bharati et al., 1986). Among these nutrients, N is the most essential for plant growth, so vast quantities of nitrogenous fertilizer are used to improve crop yields (Tegeder and Masclaux-Daubresse, 2018).

Most plants need to uptake N from the soil and water via plant roots as inorganic ions, ammonium (NH_4_^+^), and nitrate (NO_3_^–^) to maintain growth and development such as leaf expansion, stem growth and production of amino acid (Masclaux-Daubresse et al., 2010; Bloom, 2015). However, legumes form symbioses with N_2_-fixing bacteria in the soil that biologically convert atmospheric N_2_ to NH_3_ for use in the plant. This symbiotic relationship generates root nodules on the host plant in which the rhizobia are found as N_2_-fixing bacteroids (Schultze and Kondorosi, 1998; Stougaard, 2000; Rentsch et al., 2007). Interestingly, such nodulation and N_2_ fixation depend on an adequate supply of both macro- and micronutrients (Smith, 1982). In particular, among the micronutrients, iron (Fe) plays an important role in nodule formation by affecting the activation of the legume host and rhizobia (Brear et al., 2013). Molybdenum (Mo) is known as an important micronutrient for biological N-fixation in soil because Mo participates biochemical redox reaction when N-fixing bacteria converts atmospheric N_2_ into ammonium-N and nitrate-N forms in nodules (Mendel and Hänsch, 2002; Alam et al., 2015). Thus, proper concentration of Mo treatment induces increased the number and weight of nodules in leguminous crops (Hashimoto and Yamasaki, 1976; Rahman et al., 2008; Togay et al., 2008; Alam et al., 2015). In the case of silicon (Si), experiments conducted in cowpea found that application of Si to cowpea promoted nodule formation and function (Nelwamondo and Dakora, 1999). However, whether Si application affects either nodule formation or root morphological traits of soybean plant is not yet known.

Si is beneficial not only for plant growth but also in conferring tolerance to abiotic and biotic stress (Guntzer et al., 2012; Kim et al., 2014, 2016; Jang et al., 2018). In Japan, slag silicate fertilizer is applied to paddy fields to increase Si uptake of rice plants because of Si-deficient soil (Ma and Takahashi, 2002). Slag is a byproduct of iron production, so it includes various inorganic nutrients, such as Si, Fe, Mo, and magnesium (Mg) (Ma and Takahashi, 2002). Since Ma et al. (2006) identified Si transfer genes in rice root, similar genes have been discovered in barley, cucumber, tomato, maize, and soybean (Ma et al., 2006; Feng et al., 2009; Mitani et al., 2009; Yamaji et al., 2012; Deshmukh and Bélanger, 2016). Despite the identification of the Si transfer genes (GmNIP2-1 and GmNIP2-2) in soybean roots, the effects of Si on nodule formation and root morphology in soybean remain unidentified (Deshmukh et al., 2013).

The root system is an essential organ for water acquisition and nutrient absorption throughout a plant’s life (Zhao et al., 2017). Moreover, roots participate in nutrient cycling and soil formation/stabilization through interaction with soil organisms (Bardgett et al., 2014; Faucon et al., 2017). Therefore, understanding of root morphological traits helps predict plant growth and development. Information about root system architecture is derived mainly under controlled conditions at early growth stages because manually measuring root traits is time-consuming, laborious, and inaccurate in a fully grown plant (Costa et al., 2001; French et al., 2009; Lobet et al., 2011). In the field, “shovelomics” is a method of field excavation of mature root crowns for analysis of root phenotypes, so shovelomics has been broadly used for breeding and quantitative genetics (Colombi et al., 2015). However, this method measures the ground nodal root (crown root) phenotypes, disregarding the internal root system and the root traits of the younger nodes, despite the overwhelming impact of these roots on plant growth (York and Lynch, 2015). This method of collecting the root data is also time-intensive.

With the advances in data analysis, image-based measurement technologies have become an invaluable detection system for measuring various plant traits, such as leaf color, leaf area index, and stem width and height (York and Lynch, 2015). Several researchers have attempted to identify root traits by high-throughput phenotyping (Leitner et al., 2014; Pace et al., 2014; Lobet et al., 2017). Depending on the target traits, various image analysis methods have been developed, such as X-ray imaging (Mooney et al., 2012), magnetic resonance imaging (MRI; van Dusschoten et al., 2016), 2-dimensional (2D) imaging (Pornaro et al., 2017), and 3-dimensional (3D) imaging (Topp et al., 2013; van Dusschoten et al., 2016). While most of these approaches can analyze root morphological traits, such as length, area, width, and angle, MRI, X-ray, and 3D imaging techniques are difficult to apply in the field because of the associated costs, so 2D image analysis has been widely used (York and Lynch, 2015; Pornaro et al., 2017). For the same reason, the current study used 2D imaging to acquire data on root morphological traits, such as surface area, length, and angle, as well as root nodule number and size in fully grown soybean plants. To analyze morphological traits, we used WinRHIZO technology, which is a root-measuring system. Despite the importance of nodules for N fixation and N utilization, there is no simple way to quantify nodules using 2D image analysis under field conditions. For these reasons, nodule number and size were determined by the deep learning-based detection and segmentation for accurate detection.

In this study, we characterized various root traits, including nodule counts and sizes, based on 2D image analysis, to examine the effect of Si fertilization using image analysis with machine learning techniques.

## Materials and Methods

### Selection of Plant Materials

Our research team previously identified the proper concentration and uptake ratio of Si, using 15 soybean cultivars (Park et al., 2019). The results confirmed that cv. Taeseon showed a higher absorbed-Si content relative to the other cultivars. Thus, cv. Taeseon was used in the current study to investigate the effect of Si on various phenotypic root traits.

### Treatment of Si Fertilizer and Sample Preparation

The field was located at the Gyeongsang National University Research Farm (35°14′N, 128°09′E) in Jinju-si, Gyeongnam, South Korea. The experiment was set up as a split-plot arrangement in a randomized complete block design, with three replications. The treatments consisted of (i) Control, (ii) Si–soil (Si applied to soil), and (iii) Si–soil + leaf (Si applied to soil and leaves) since Si is known to be absorbed via leaves as well (Cao et al., 2020). Each plot size was 4 m × 4 m, with 1 m row spacing. Before the planting, ridges (0.3 m high × 0.7 m wide) were prepared for each plot. Soybean seeds were sown on June 15, 2018, at a planting distance of 0.15 m using a disk hand planter (TP-10RA, AGRITECNO YAZAKI Co., Ltd., South Korea). For Si soil application, we applied 1.6 kg of commercial silicate fertilizer (25% SiO_2_–2% MgO–40% CaO; Pungnong Co., Ltd., South Korea) to the soil surface, according to the manufacturer’s recommendations (100 kg/1000 m^2^), at planting. Additionally, for foliar application of Si (Si–soil + leaf), we sprayed 2.0 mM of sodium metasilicate (Na_2_SiO_3_; Sigma-Aldrich, United States) when the 4th–5th trifoliate leaf had fully emerged and unrolled (V4–V5 stage). To determine the soil chemical properties in the research area, 20 samples were taken from 0 to 30-cm depth, then air-dried. The soil at the experimental site contained organic matter, available phosphate, K, Ca, and Mg at 9.3 g/kg, 55 mg/kg, 0.25 cmol/kg, 4.07 cmol/kg, and 0.47 cmol/kg, respectively (Table 1).

**TABLE 1:** Soil chemical properties of the experimental site (0–30 cm).

pH	EC	OM	Av. P_2_O_5_	K	Ca	Mg	ORD
1:5	1:5 (dS/m)	(g/kg)	(mg/kg)	———– (cmol_*c*_/kg) ———–			(kg/10a)
6.65	0.21	9.3	55.0	0.25	4.07	0.47	133.00

### Phenotypic Data Collection

#### Root Sampling and Analysis

**FIGURE 1:**
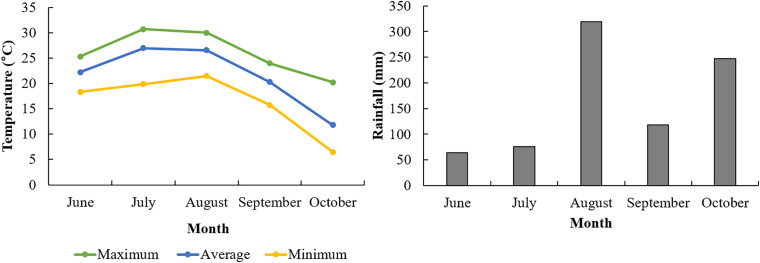
Weather condition of experimental field in 2018.

We applied three different Si applications as seen in “Materials and Methods” to identify Si effects on root morphology. We collected 30 root samples from each plot when the soybean plants reached the R8 stage, i.e., when 95% of the pods display full mature color, and when root growth has ceased (Nleya et al., 2013). Before sampling, the above-ground plant parts were removed, then a circle was marked by a round-basket (30 cm in diameter) on the soil around the target plant. The target root and soil were carefully removed to a depth of 30 cm. To minimize root and nodule loss, roots were gently washed with tap water which was contained in a basket (width 304 mm × height 330 mm) to remove attached soil. Weather conditions during the entire soybean growth period is described in Figure 1. The average temperature was 11.8°C (October) −26.9°C (July) and the rainfall showed 64.0 mm (June) −319.5 mm (August) (Figure 1).

#### Image Analysis for Root Phenotype

Root images were acquired using a mirrorless camera (M100, Canon, Japan) with a mini-rhizobox (Supplementary Figure S1). The camera information is detailed in Supplementary Table S1. All root samples were imaged with the lens focus fixed at 22 mm. Additionally, to measure root morphological traits, the collected root images were analyzed using WinRHIZO Pro software (WinRHIZO, Regent Instruments, Inc., Canada). Each root trait is defined in Supplementary Table S2.

#### Image Analysis for Nodule Count and Size

**FIGURE 2:**
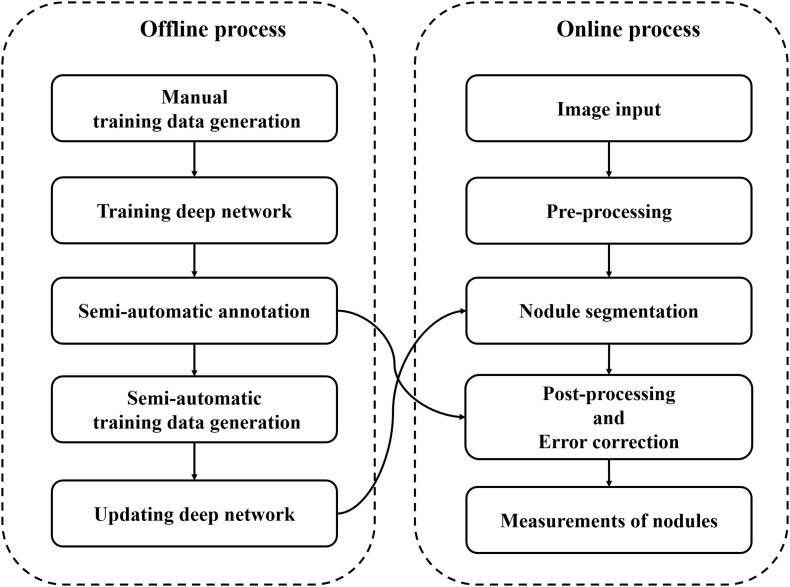
Image analysis pipeline.

Almost all nodules of soybean roots were small and overlapped each other. The measurements (e.g., number of objects, areas of an object) of small and overlapping objects were computer vision challenges. Therefore, we designed a pipeline of accurate measurements by using a deep network-based nodule segmentation and semi-automatic annotation function-based error correction process (Figure 2). The pipeline code is available at https://github.com/ektf1130/nodule_in_soybean_root. The details are provided below.

##### Training deep network and nodule segmentation

**FIGURE 3:**
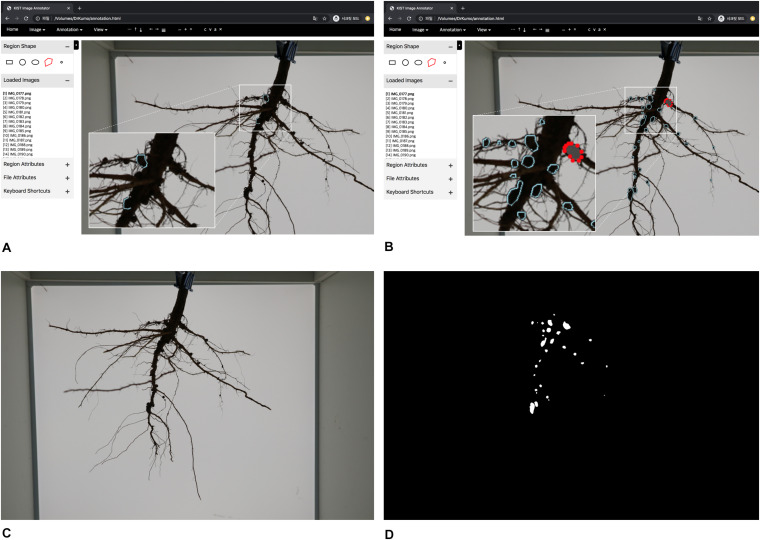
Graphical User Interface of annotation tool and training data generation. **(A)** overview of annotation tool **(B)** manual inputs of annotation by clicking polygons **(C)** an RGB color image **(D)** a mask image.

U-net architecture is a robust package for segmentation of thin and small objects (Olaf et al., 2015). It is a convolutional network architecture for fast and precise segmentation of images known to be the prior best method for segmentation (Livne et al., 2019). Thus, U-net was used for pixel-wise segmentation of soybean root nodules, with separation of the nodules in a soybean root from the background. To train the segmentation network, RGB color images and their mask images are required; the mask image designates the foreground nodules as white and the background as black (Figures 3C,D). U-Net is used mainly to input the image dimension as multiples of 32, and our network used 1024 × 1024 pixel images as inputs for maximum resolution considering the specifications of our training computer; Intel(R) Core(TM) i7-4790K CPU @ 4.00 GHz, 32 GB RAM, NVIDIA GeForce GTX TITAN X 12 GB.

**FIGURE 4:**
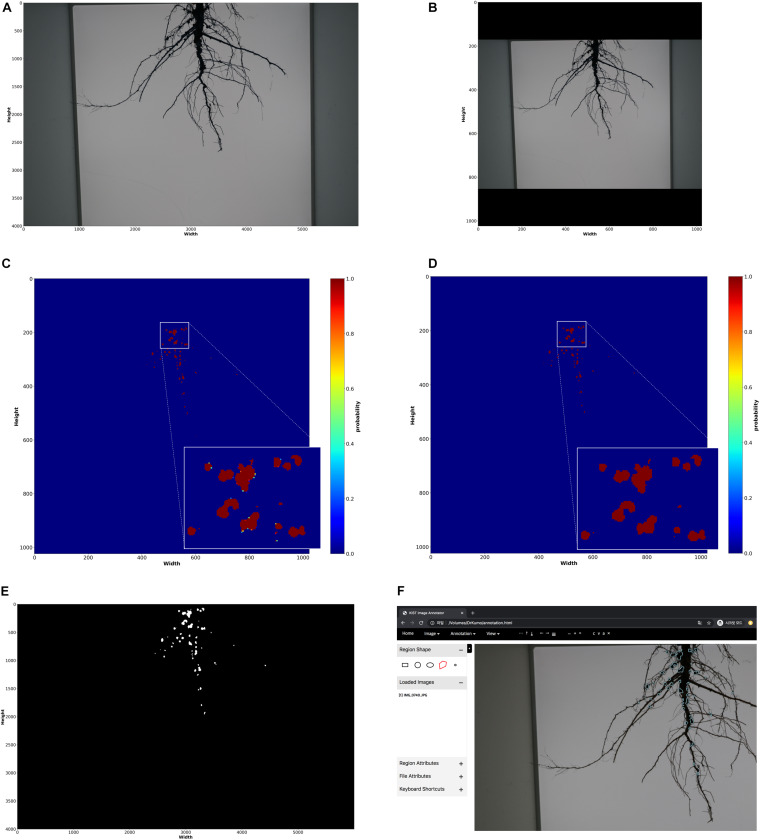
Nodule measurement processes. **(A)** raw input RGB color image (6000 × 4000 dimensions) **(B)** pre-processed RGB color image (1024 × 1024 dimensions) **(C)** segmentation results; probability for each pixel (1024 × 1024 dimensions) **(D)** post-processing results on segmentation result (1024 × 1024 dimensions) **(E)** reconstruction of original size (6000 × 4000 dimensions) **(F)** loading nodule regions on annotation tool; the regions extracted from the final reconstructed segmentation result is displayed on the tool.

In the offline process shown as Figure 2, initially, we created 35 masks (annotations) of root images manually using our customized annotation tool. The RGB color and mask images (i.e., training data) were properly scaled and padded for the network size by pre-processing; the original size of RGB and mask images (6000 × 4000 pixels) were converted to 1024 × 1024 pixel images (Figures 4A,B). Then, our deep network was trained using the processed training data.

In the online process, input root RGB color images are converted into 1024 × 1024 pixel images, and nodule segmentation of the root is performed using the trained deep network; the network outputs mask images.

##### Post-processing and Nodule measurements

**FIGURE 5:**
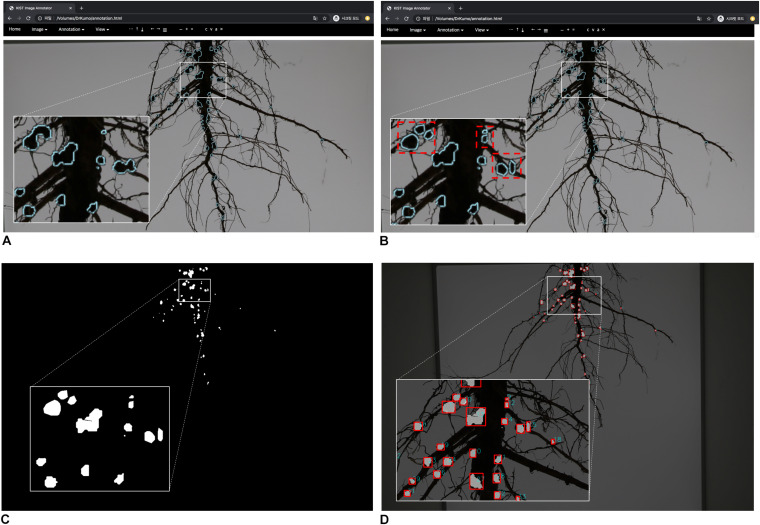
Semi-automatic annotation function-based error correction. **(A)** confirm of error nodules **(B)** error correction; red rectangles **(C)** segmentation result (mask image) before error correction **(D)** segmentation result after error correction.

The fully connected conditional random fields (Krähenbühl and Koltun, 2011) was mainly used to remove noises from mask images (segmentation results) generated by the trained deep network as a post-processing step, to improve the accuracy of segmentation at the pixel level. As a result of the nodule segmentation, the mask image had probabilities of between 0 and 1 for each pixel (Figure 4C). Krähenbühl and Koltun (2011) used the probabilities, and color and position of each pixel to remove noise (Figure 4D). After noise removal, the holes representing nodules in the mask images were filled using the closing process (Figure 4E). Then, the semi-automatic annotation-based error correction was performed. Finally, the fully connected components were detected, and noise was removed by component sizes. The nodules were counted using the detected components (i.e., blobs), shown in Figure 5D, and the nodule size was converted from pixels to actual size (i.e., mm^2^) using a reference size; the actual sizes of the reference were measured in advance.

##### Semi-automatic annotation-based error correction and transfer learning

In general, adding more validated training data increases the performance of the deep learning-based segmentation network and widens the range of data that can be processed. However, generating annotation images (training data) is labor-intensive; it is time-consuming to generate training data because nodules in a root are small and numerous, and the amount of training data required for optimal deep network performance is unknown. Also, even with the optimal trained networks, segmentation errors can occur for new root images. Therefore, we created a semi-automatic annotation tool to improve the mentioned problem. Our tool was customized based on the existing tool that has a simple to use and convenient graphical user interface (GUI) to generate training data (Dutta et al., 2016). We have added a function that automatically annotates regions of nodules using our pre-trained deep network on the existing tool. Our customized tool was used for semi-automatic annotation (generating training data) and error correction.

Initially, a pre-trained deep network is required for the semi-automatic annotation. So, we manually generated an appropriate amount of training data (mask images) using the customized tool; the appropriate amount means minimal labor for manual annotation. We created 35 training data sets of root images manually; there are multiple nodules on each root, and we drew polygons by clicking on the contour of each nodule (Figures 3A,B). Then, a deep network was trained using the training data; it is a pre-trained deep network. The nodule segmentation of the pre-trained deep network was not robust, but it was enough to use as an aid to create additional training data (Supplementary Table S3).

**FIGURE 6:**
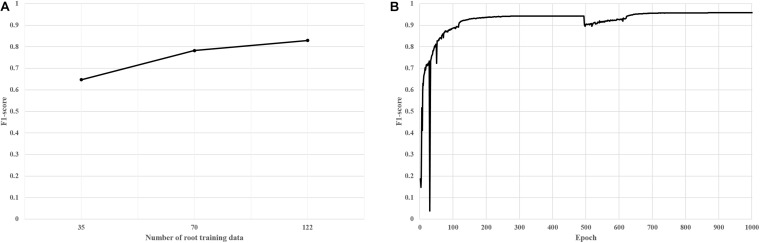
F1-scores of trained deep segmentation networks; *y*-axis of 1.0 means 100 percentage matched between predicted mask image and actual mask image (ground truth image). **(A)** F1-scores of trained networks according to the number of training data **(B)** F1-scores of final deep segmentation network; an epoch means an iteration of the number of training data.

New root images were input into the pre-trained network and mask images were created. Next, the coordinates of contours of each nodule were computed from the mask images, and were then imported into the customized tool and displayed on the new root images (Figures 4E,F). We then manually corrected the error regions of the contours using the GUI tool (Figures 5A,B); the semi-automatic annotation processes significantly reduced the time to create additional training data, which were used to update the pre-trained deep network (i.e., transfer learning). The segmentation performance of each updated pre-trained deep network was improved by incorporating additional training data (Figure 6A). We repeated these processes until the segmentation performance of the updated pre-trained deep network was sufficiently robust. The performance was evaluated using the F1-score metric, which is frequently used to evaluate segmentation performance (Goutte and Gaussier, 2005; Csurka et al., 2013). The F1-score can be used to evaluate our pre-trained network without bias because the score was considered a balance between precision and recall.

$$P\,r\,e\,c\,i\,s\,i\,o\,n\,\left(P\right)=\frac{T\,P}{T\,P+F\,P}$$

$$R\,e\,c\,a\,l\,l\,\left(R\right)=\frac{T\,P}{T\,P+F\,N}$$

$$F\,1\,s\,c\,o\,r\,e=2\times \frac{P\times R}{P+R}$$

True Positive (TP), False Positive (FP), and False Negative (FN) are ratios between the predicted value (nodule and background) from the pre-trained deep network and the actual value (mask images); TP means that the actual foregrounds are predicted as foregrounds by the pre-trained network; FP means that the actual backgrounds are predicted as foregrounds by the network; and FN means that the actual foregrounds are predicted as backgrounds. Finally, the pre-trained deep network using 135 training data was created and showed robust performance according to the F1-score (Figure 6B).

This pre-trained network became the final nodule segmentation network, with fewer errors than the initial pre-trained network. Additional error correction was performed with the same process as the initial semi-automatic annotation mentioned above. In the online process, error correction was performed on the mask image of the post-processing result, and the new mask was generated after error correction. Nodule measurements were then obtained using the corrected mask images (Figures 5C,D).

### Statistical Analysis

A randomized block design was used with subsampling and three replications (blocks). Three treatments (fertilizer type) were applied randomly within each block. Analysis of variance (ANOVA) models were used to investigate the effect of the Si treatments on the root traits. For these traits, the data tidying consisted of computing the sum of nodule size for each image and converting the area measured in millimeters squared to centimeters squared, resulting in 270 data points. The statistical model is given by:

$$y_{i\,j\,k}=\mu +\beta_{j}+\alpha_{i}+\delta_{i\,j}+{}_{i\,j\,k}$$

where *y*_*ijk*_ is the response variable (phenotype); μ is the intercept; β_*j*_ is the *j*-th block effect; α_*i*_ is the *i*-th treatment effect; δ_*ij*_ is the *ij*-th experimental error (plot effect), and ∼i⁢j⁢kN(0,σ2) is the sampling error.

A quantile–quantile plot was performed to verify the null hypothesis of the normality of the residuals, and the fitted versus residuals plot was tested for homogeneity of variance. For some phenotypes (nodule size and count), logarithmic transformation was sufficient to deal with non-normality and heteroscedasticity. For others, a heteroscedastic linear mixed model was fitted to verify the null hypothesis (i.e., no difference between treatments). The variance structure used to model heteroscedasticity was proportional to the power of the absolute value of the fitted values or different variances across experimental units. Tukey’s test was used to compare the means of the treatments. Correlations between root traits in which the treatments were significant, were computed.

## Results

**FIGURE 7:**
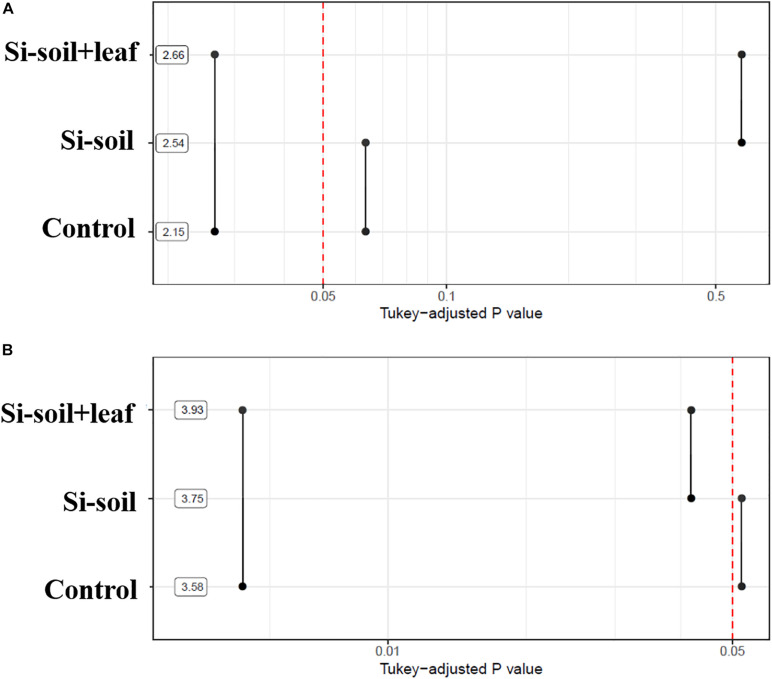
*P*-values associated with pairwise comparisons of means of treatments for nodules size **(A)** and nodules counts **(B)**. Means are shown using a logarithmic scale.

Significant differences in nodule size and number were detected among the treatments (Figure 7). The Si–soil + leaf plants showed a significantly higher mean nodule size as compared with control and Si-soil treated plants. For the number of nodules, the only differences were between Si–soil + leaf and control and between Si–soil and Si–soil + leaf. Overall, Si treatments were effective on increased nodule size and number when applied to both soil and leaf at the same time.

**FIGURE 8:**
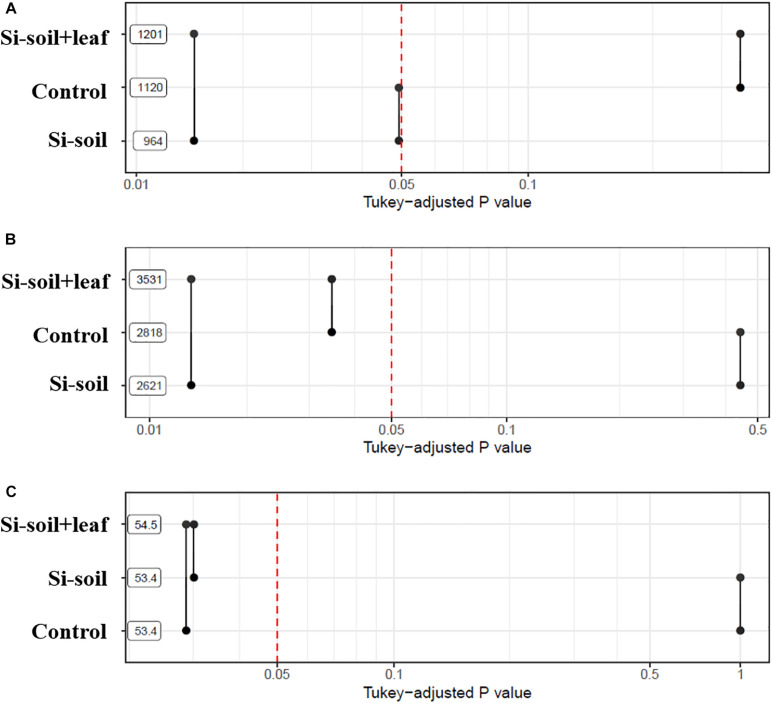
*P*-values associated with pairwise comparisons of means of treatments for length **(A)**, number of forks **(B)**, and average link angles **(C)**.

To investigate if the root architecture phenotypes were affected by Si treatment, nine aspects of root structure were evaluated, including length, average diameter, number of tips, number of forks, projected area, main total length, lateral total length, link projected area, link average length, link average surface area, link average diameter, and link average branching angles (Supplementary Table S2). Among them, three variables, namely length, number of forks, and link average branching angles, showed significant differences (Table 2). This implies that length, the number of forks, and average link angles are important morphological traits for Si response in soybean root. Length showed significant difference between Si–soil and control, and between control and Si–soil + leaf (Figure 8A). However, our results did not show significant difference between Si–soil and control in number of forks, while a significant difference observed in comparison with control and Si–soil + leaf, and between Si–soil and Si–soil + leaf (Figure 8B). Link branching angle observed similar pattern with number of forks. Except for comparison with control and Si–soil, all treatment showed significant difference at *P* < 0.05 (Figure 8C). Those three root traits were then correlated with the nodule size and number of nodules formed in the control and treated plants to determine if the root structure phenotypes affected nodule formation (Table 3). Treatments do not have effects between nodule size and the number of nodules and between root length and the number of forks. However, there were high correlations (*r* = 0.95–1.00) between nodule size and number in the control and all treatments, indicating that nodule size and number are highly associated, irrespective of the amount of Si the plant absorbs. There were also high correlations (*r* = 0.92–1.00) between number of forks and length in the control and all treatments, suggesting that fork development, as the initiation point of elongation of lateral roots, augments root length at all Si levels. There were significant Si treatment effects on the correlation between average branching angle and root length and between average branching angle and number of forks, with correlation values ranging from *r* = 0.92 to *r* = 0.99, showing that Si is responsible for lateral root formation and its angle. The correlation between nodule size and between average branching angle and between nodule number and average branching angle was highly affected only by the Si–soil + leaf, although there was a low correlation (*r* = 0.32) between nodule size and average branching angle in the control plants. However, the correlation results between nodule size and root length, between nodule size and number of forks, between nodule number and root length, and between nodule number and number of forks were difficult to interpret because the correlations were high both in the control and in the Si treatment on Si–soil + leaf treatment, while the correlation was low in the Si–soil treatment.

**TABLE 2:** *F* test for fixed effects from the mixed model fitted to root architecture data originated from a randomized complete block design with subsampling.

Variable	F statistics	*P*-value
Length	14.79	0.01
Area projection	4.10	0.11
Average diameter	0.98	0.45
Number of tips	3.44	0.14
Number of forks	14.12	0.02
Linked average surface area	1.32	0.36
Linked average diameter	1.26	0.38
Average link angles	11.80	0.02
Main total length	0.28	0.77

**TABLE 3:** Correlation network plot between phenotypic data obtained by image analysis and root architecture data.

	Number of nodules	Root length	Number of forks	Average link angle
Nodule size	0.95a***	0.89a***	0.97a***	0.32a**
	1.00b**	0.28b**	0.38b***	0.11b
	0.98c**	0.98c**	0.99c***	0.98c***
Number of nodules	1	0.99a***	0.97a***	0.00a
		0.25b*	0.35b***	0.08b
		1.00c***	1.00c***	0.92c***
Root length		1	0.92a***	^a^−0.14
			0.99b***	0.99b**
			1.00c***	0.92c***
Number of forks			1	0.26a*
				0.96b***
				0.94c***

^*a*^Control. ^*b*^Si–soil. ^*c*^Si–soil + leaf. *, **, and ***, *P* < 0.05, 0.01, and 0.001, respectively.

## Discussion

The image was obtained to maximize the area of roots by adjusting the camera angle. Nonetheless, it is impossible to characterize the nodule behind the roots image taken for this experiment. However, it is still analyzable because all treatments including the control had the same method for image obtaining. In other words, if one image has error, the others would have same error. And this is why a lot of images were taken replications to reduce error. Considering this, the high contrast image was fine to characterize nodule. The way the nodule was characterized was to detect the bumpy area from linear line of roots. Based on this, the nodule was found and counted. Once nodule was detected, the part of circle could be detected. Then, based on this part of circle, the rest of the nodule was inferred. According to our results, Si treated soybean plant showed increased nodule number and nodule size as compared to non-Si treated soybean plant. This result revealed that Si treatment induces or participates nodule formation. We assumed that the reason seems to several reasons. The first hypothesis is that Si application is responsible for ABA accumulation, which, in turn, causes lateral root formation and growth, increasing the number of infection sites for nodulation. Furthermore, the increased root angle associated with Si treatment was highly correlated with nodule formation. It may worth to investigate if it is true or not by measuring ABA in the future.

The second hypothesis is that Si influence on the expression of *nod* genes which affect to formation of nodules in leguminous plants (Nelwamondo and Dakora, 1999). Furthermore, Si is involved in synthesis of isoflavonoids, thus application of Si fertilizer into soil induces increased nodule formation of leguminous plants (van Bockhaven et al., 2013). Because, leguminous plants release isoflavonoids for enticing nitrogen-fixing bacteria (Eckardt, 2006).

Si is known to increase nodule formation and to elongate root length in legumes, which increases the number of potential sites for infection by rhizobial invasion (Nelwamondo and Dakora, 1999). Accordingly, the results in the current study were consistent with the findings of Nelwamondo and Dakora (1999). Root length was highly correlated with nodule size and number in the current study. Furthermore, Si seems to be involved with the total number of secondary roots as forks (Guo et al., 2006). We assumed that this phenomenon also was due to the accumulation of abscisic acid (ABA) in roots, as a result of the Si treatment (Signora et al., 2001; Dakora and Nelwamondo, 2003). According to this study, ABA accumulation, due to Si application, influences not only root growth but also lateral root development and growth. Liang and Harris (2005) likewise reported that ABA stimulates lateral root formation in legumes.

In addition, lateral root formation increases as the concentration of ABA increases (Liang and Harris, 2005). Previously, nodule number was responsive to Si supply in legumes (Nelwamondo and Dakora, 1999) because secondary root formation is highly responsive to the Si concentration (Guo et al., 2006). Together, these observations could explain why Si–soil + leaf treatment showed a stronger correlation than Si–soil. In other words, the Si concentration may not be sufficient to be influential to lateral root formation. Thus, it would be worthwhile to investigate the optimal concentration of Si for nodulation and each of the root morphological traits.

Nodules at a depth of 20 cm or greater were frequently observed at different sites in different cultivars (Grubinger et al., 1982). However, the number of nodules varied significantly in the vertical distribution, depending on the cultivar. If a certain depth is advantageous for nodule formation, the root structure could be influential to nodule formation as well. In the current study, the Si treatment was highly correlated with a higher root angle, which means that Si was involved with root formation horizontally, near the soil surface, and consequently, there was more nodule formation near the soil surface. This result might indicate that the cultivar and bacteria used in this study were a lot more influential in promoting nodule formation in the near soil surface.

The reason for the high correlations between nodule size and root length, between nodule size and forks number, between nodule number and root length, and between nodule number and forks number in both the control and Si–soil + leaf but low correlations for Si–soil could be because of the huge variation found in the data set in the current study (Table 4). There are very significant differences among blocks for nodule size and the number of nodules, which are larger effects than those among treatments. Consequently, it is hard to detect clear difference. This could be because soybean plants are very susceptible to various abiotic stresses (Board, 2013). It may be enhanced with more sophisticated experimental design.

**TABLE 4:** Analysis of variance from randomized complete blocks design with subsampling for nodule size (mm^2^) and nodule counts.

Source	Mean square	
	Nodule size	Nodule number
Blocks	16.49**	22.01***
Treatments	6.28*	2.75**
Experimental error	0.61	0.10
Sampling error	0.21	0.15

*, **, and ***, *P* < 0.05, 0.01, and 0.001, respectively.

The robust measurements in the current study using the emerging, deep learning-based detection and segmentation (Olaf et al., 2015; Ren et al., 2017; Chen et al., 2018; Feng et al., 2020; Gao et al., 2020) allowed distinct separation of the object from the background, unlike the conventional image processing methods, which are not robust to various types of objects and noises (Khan et al., 2010; Wang et al., 2011). Overall, the image analysis using machine learning in the current study enabled us to characterize numerous nodules in roots in many plants, which is truly huge advance.

## Conclusion

Using 2D images, we analyzed huge number of soybean root nodule by machine learning technology. Thus we identified Si application induce increase of nodule size and number. However, current results cannot prove the accurate mechanism. We assumed two possibility. The first hypothesis is that Si application accumulate hormone ABA thus, this phenomena induce various root responses such as nodule formation and root architecture. The second hypothesis is that Si treatment not only stimulate nod gene but also increase isoflavonoid contents, consequently, increased nodule number and size occurs. To prove those hypothesis, additional experiments are required. Furthermore, comparative experiments are need for confirming the utility of nodule baseline technology in leguminous plants. If we get reasonable result, we will open website to use those technology to other researcher.

## Data Availability Statement

The datasets generated for this study are available on request to the corresponding author.

## Author Contributions

YC and SH wrote the manuscript and analyzed the data. UL participated in deep learning analysis. RS statistically analyzed the data. C-IN assisted with the data and sample collection. YK inspected the experimental design and revised the manuscript. All authors contributed to the article and approved the submitted version.

## Conflict of Interest

The authors declare that the research was conducted in the absence of any commercial or financial relationships that could be construed as a potential conflict of interest.

## Supplementary Material

Supplementary Figure 1 Mini-rhizobox for image acquisition.

Supplementary Table 1 Camera specifications.

Supplementary Table 2 Short description of the parameters analyzed in the study using WinRHIZO root analysis software (Regent Instruments, Canada).

Supplementary Table 3 Training result of nodule size and number of nodule. Deep network was trained using 35 training data set and then, the test was conducted by using 200 root images.
