# Supplementary figures and images for: Image-Based Machine Learning Characterizes Root Nodule in Soybean Exposed to Silicon
Source: Front Plant Sci. 2020 Oct 28;11:520161. doi: 10.3389/fpls.2020.520161 (PMC7655541; doi:10.3389/fpls.2020.520161)

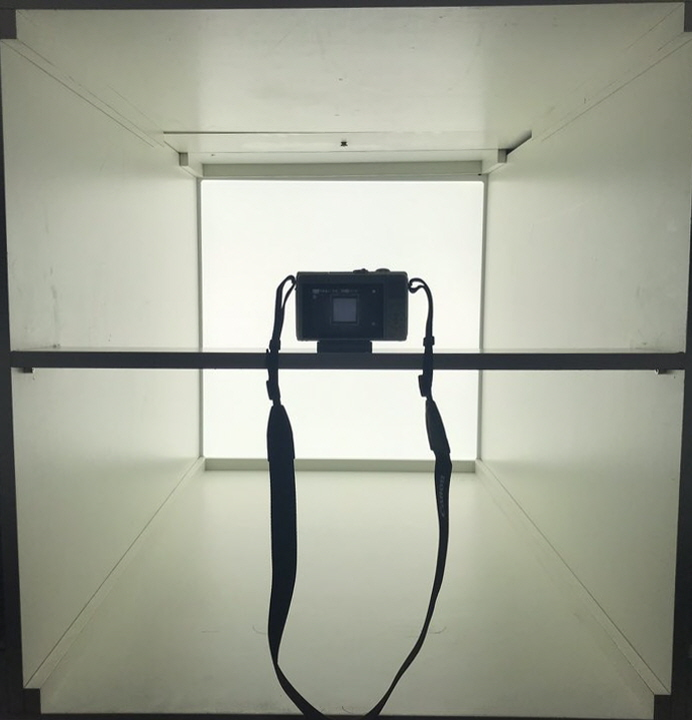

Supplement: Supplementary Figure 1 — Mini-rhizobox for image acquisition. [file Image_1.TIF]
