# Supplementary material for: Image-Based Machine Learning Characterizes Root Nodule in Soybean Exposed to Silicon
Source: Front Plant Sci. 2020 Oct 28;11:520161. doi: 10.3389/fpls.2020.520161 (PMC7655541; doi:10.3389/fpls.2020.520161)
Supplement: Supplementary Table 1 — Camera specifications. [file Table_1.DOCX]

**Supplementary Table S1** Camera specifications

| Dimensions (H × W × D) | 108.2 × 67.1 × 35.1 mm |
| --- | --- |
| Weight | 302 g (including battery and memory card) |
| Image sensor | 22.3 × 14.9 mm CMOS (24.2 MP) |
| Image processor | DIGIC 7 |
| Lens | EF-M 11–22 mm F4-5.6 IS STM |
