# Supplementary material for: Image-Based Machine Learning Characterizes Root Nodule in Soybean Exposed to Silicon
Source: Front Plant Sci. 2020 Oct 28;11:520161. doi: 10.3389/fpls.2020.520161 (PMC7655541; doi:10.3389/fpls.2020.520161)
Supplement: Supplementary Table 2 — Short description of the parameters analyzed in the study using WinRHIZO root analysis software (Regent Instruments, Canada). [file Table_2.DOCX]

**Supplementary Table S2** Short description of the parameters analyzed in the study using WinRHIZO root analysis software (Regent Instruments, Canada)

| **Parameter** | **Description** |
| --- | --- |
| Length | Length of root skeleton, counting pixels while taking into account the direction of displacement of the root |
| Average diameter | Average diameter of roots |
| Tip | Number of root endings |
| Fork | Number of new branching incidences |
| Projected area | Area occupied by roots in the image. Counting the number of pixels belonging to the root in the detected root images, then multiplying the value by the pixel area |
| Main total length | Total length of links of order 0 (main) |
| Lateral total length | Total length of links of order 1 (lateral) |
| Link projected area | Sum of projected areas for links that belong to the order |
| Link average length | A link is a root part between two forks or a fork and a tip. Average length of links that belong to the order |
| Link average surface area | A link is a root part between two forks or a fork and a tip. Average surface area for links belong to the order |
| Link average diameter | A link is a root part between two forks or a fork and a tip. Average of the average diameters for links that belong to the order |
| Link average branching angle | A link is a root part between two forks or a fork and a tip. Average link angles for links belong to the order |
