# Supplementary material for: Image-Based Machine Learning Characterizes Root Nodule in Soybean Exposed to Silicon
Source: Front Plant Sci. 2020 Oct 28;11:520161. doi: 10.3389/fpls.2020.520161 (PMC7655541; doi:10.3389/fpls.2020.520161)
Supplement: Supplementary Table 3 — Training result of nodule size and number of nodule. Deep network was trained using 35 training data set and then, the test was conducted by using 200 root images. [file Table_3.DOCX]

**Supplementary Table S3** Training result of nodule size and number of nodule. Deep network was trained using 35 training data set and then, the test was conducted by using 200 root images.

| Genotypes | Number of nodule | | Nodule size | |
| --- | --- | --- | --- | --- |
|  | Annotation (number) | Prediction (number) | Annotation  (mm^2^) | Prediction (mm^2^) |
| 1 | 51 | 41 | 1266 | 1208 |
| 2 | 63 | 51 | 1530 | 1510 |
| 3 | 130 | 96 | 5332 | 5114 |
| 4 | 100 | 71 | 3414 | 3026 |
| 5 | 89 | 64 | 2272 | 2266 |
| 6 | 83 | 59 | 1948 | 1856 |
| 7 | 53 | 52 | 842 | 782 |
| 8 | 53 | 41 | 1276 | 1132 |
| 9 | 60 | 55 | 1546 | 1614 |
| 10 | 49 | 61 | 2504 | 2420 |
| 11 | 80 | 85 | 2614 | 2436 |
| 12 | 111 | 79 | 3472 | 3188 |
| 13 | 82 | 58 | 1602 | 1516 |
| 14 | 88 | 65 | 1168 | 1082 |
| 15 | 97 | 50 | 2036 | 1928 |
| 16 | 86 | 64 | 1968 | 1742 |
| 17 | 73 | 54 | 3428 | 3212 |
| 18 | 113 | 61 | 1326 | 1012 |
| 19 | 31 | 38 | 966 | 922 |
| 20 | 62 | 39 | 750 | 558 |
| 21 | 11 | 13 | 116 | 84 |
| 22 | 99 | 61 | 2188 | 1848 |
| 23 | 47 | 42 | 518 | 420 |
| 24 | 41 | 45 | 1084 | 1052 |
| 25 | 13 | 14 | 102 | 84 |
| 26 | 66 | 73 | 1144 | 1068 |
| 27 | 12 | 14 | 130 | 128 |
| 28 | 26 | 24 | 152 | 128 |
| 29 | 39 | 40 | 706 | 694 |
| 30 | 35 | 36 | 454 | 390 |
| 31 | 87 | 64 | 1312 | 1304 |
| 32 | 19 | 17 | 370 | 342 |
| 33 | 25 | 31 | 424 | 462 |
| 34 | 38 | 33 | 836 | 838 |
| 35 | 93 | 71 | 3422 | 3656 |
| 36 | 44 | 34 | 1726 | 1728 |
| 37 | 73 | 63 | 2736 | 2426 |
| 38 | 35 | 19 | 854 | 688 |
| 39 | 100 | 79 | 2488 | 2176 |
| 40 | 68 | 52 | 1482 | 1282 |
| 41 | 43 | 37 | 1252 | 1080 |
| 42 | 41 | 22 | 410 | 294 |
| 43 | 54 | 55 | 1300 | 1044 |
| 44 | 33 | 39 | 642 | 514 |
| 45 | 140 | 109 | 4854 | 4212 |
| 46 | 20 | 13 | 240 | 164 |
| 47 | 125 | 84 | 3110 | 2962 |
| 48 | 46 | 35 | 848 | 720 |
| 49 | 89 | 64 | 2318 | 2292 |
| 50 | 26 | 31 | 544 | 516 |
| 51 | 51 | 34 | 896 | 850 |
| 52 | 35 | 33 | 828 | 838 |
| 53 | 37 | 39 | 814 | 828 |
| 54 | 102 | 79 | 2726 | 2744 |
| 55 | 50 | 48 | 1028 | 1118 |
| 56 | 15 | 17 | 84 | 96 |
| 57 | 115 | 86 | 2482 | 2352 |
| 58 | 47 | 39 | 1148 | 1052 |
| 59 | 79 | 54 | 1110 | 856 |
| 60 | 37 | 37 | 620 | 514 |
| 61 | 68 | 56 | 2236 | 1880 |
| 62 | 38 | 34 | 548 | 416 |
| 63 | 27 | 21 | 1174 | 1006 |
| 64 | 45 | 47 | 1522 | 1414 |
| 65 | 60 | 31 | 1910 | 1570 |
| 66 | 40 | 40 | 1564 | 1372 |
| 67 | 18 | 20 | 154 | 134 |
| 68 | 11 | 11 | 56 | 44 |
| 69 | 53 | 42 | 1266 | 1170 |
| 70 | 44 | 25 | 652 | 662 |
| 71 | 26 | 17 | 448 | 382 |
| 72 | 31 | 42 | 1498 | 1520 |
| 73 | 82 | 65 | 3276 | 3314 |
| 74 | 83 | 64 | 2414 | 2566 |
| 75 | 88 | 64 | 2148 | 2204 |
| 76 | 68 | 51 | 914 | 912 |
| 77 | 151 | 118 | 3766 | 3362 |
| 78 | 26 | 21 | 474 | 374 |
| 79 | 32 | 26 | 530 | 402 |
| 80 | 17 | 15 | 184 | 150 |
| 81 | 52 | 43 | 914 | 612 |
| 82 | 75 | 61 | 2100 | 1632 |
| 83 | 120 | 94 | 1886 | 1536 |
| 84 | 60 | 55 | 1720 | 1480 |
| 85 | 75 | 46 | 1718 | 1408 |
| 86 | 93 | 91 | 3222 | 3014 |
| 87 | 113 | 85 | 3394 | 3350 |
| 88 | 122 | 86 | 3478 | 3376 |
| 89 | 83 | 97 | 2252 | 2264 |
| 90 | 24 | 22 | 420 | 392 |
| 91 | 120 | 97 | 3192 | 3278 |
| 92 | 80 | 62 | 2006 | 1872 |
| 93 | 97 | 84 | 3594 | 3578 |
| 94 | 44 | 35 | 1238 | 1226 |
| 95 | 52 | 43 | 1040 | 1018 |
| 96 | 36 | 23 | 1158 | 1132 |
| 97 | 98 | 69 | 1606 | 1392 |
| 98 | 90 | 70 | 2308 | 2178 |
| 99 | 105 | 80 | 3368 | 2712 |
| 100 | 62 | 54 | 1634 | 1442 |
| 101 | 68 | 37 | 1306 | 972 |
| 102 | 97 | 80 | 3030 | 2662 |
| 103 | 27 | 23 | 1336 | 1232 |
| 104 | 102 | 73 | 3450 | 3304 |
| 105 | 41 | 45 | 1728 | 1732 |
| 106 | 44 | 56 | 656 | 540 |
| 107 | 104 | 68 | 2054 | 1964 |
| 108 | 27 | 28 | 316 | 290 |
| 109 | 61 | 52 | 1512 | 1710 |
| 110 | 23 | 28 | 626 | 634 |
| 111 | 40 | 27 | 452 | 384 |
| 112 | 79 | 75 | 4460 | 4640 |
| 113 | 138 | 93 | 2990 | 2728 |
| 114 | 52 | 43 | 592 | 478 |
| 115 | 69 | 52 | 1726 | 1696 |
| 116 | 88 | 61 | 1756 | 1548 |
| 117 | 83 | 67 | 2752 | 2550 |
| 118 | 48 | 42 | 1336 | 1108 |
| 119 | 46 | 33 | 948 | 738 |
| 120 | 46 | 36 | 462 | 348 |
| 121 | 117 | 83 | 3732 | 3376 |
| 122 | 17 | 12 | 140 | 84 |
| 123 | 18 | 13 | 190 | 120 |
| 124 | 78 | 70 | 3332 | 3042 |
| 125 | 47 | 39 | 820 | 624 |
| 126 | 106 | 76 | 4280 | 4092 |
| 127 | 84 | 78 | 3260 | 3334 |
| 128 | 39 | 31 | 1468 | 1520 |
| 129 | 118 | 81 | 2912 | 2756 |
| 130 | 51 | 45 | 1082 | 1076 |
| 131 | 31 | 29 | 364 | 336 |
| 132 | 84 | 79 | 3336 | 3426 |
| 133 | 77 | 76 | 2248 | 2288 |
| 134 | 54 | 35 | 1186 | 1028 |
| 135 | 89 | 63 | 2060 | 1774 |
| 136 | 76 | 60 | 1622 | 1416 |
| 137 | 141 | 100 | 3460 | 3294 |
| 138 | 64 | 47 | 1780 | 1540 |
| 139 | 44 | 36 | 1100 | 938 |
| 140 | 47 | 36 | 1916 | 1716 |
| 141 | 64 | 70 | 1418 | 1268 |
| 142 | 62 | 52 | 1128 | 876 |
| 143 | 82 | 75 | 1458 | 1302 |
| 144 | 19 | 20 | 222 | 186 |
| 145 | 102 | 87 | 3694 | 3636 |
| 146 | 21 | 21 | 178 | 182 |
| 147 | 106 | 89 | 3306 | 3572 |
| 148 | 36 | 29 | 830 | 900 |
| 149 | 25 | 31 | 240 | 258 |
| 150 | 28 | 32 | 362 | 382 |
| 151 | 37 | 27 | 388 | 274 |
| 152 | 26 | 21 | 182 | 120 |
| 153 | 99 | 71 | 1456 | 1140 |
| 154 | 88 | 63 | 980 | 872 |
| 155 | 51 | 63 | 986 | 946 |
| 156 | 23 | 14 | 468 | 438 |
| 157 | 44 | 38 | 1024 | 810 |
| 158 | 136 | 97 | 3616 | 3036 |
| 159 | 51 | 49 | 1370 | 1136 |
| 160 | 38 | 31 | 694 | 588 |
| 161 | 79 | 65 | 2190 | 1952 |
| 162 | 103 | 67 | 3796 | 3716 |
| 163 | 45 | 33 | 1188 | 1092 |
| 164 | 38 | 33 | 960 | 926 |
| 165 | 109 | 81 | 1806 | 1690 |
| 166 | 72 | 61 | 1008 | 1046 |
| 167 | 34 | 33 | 1080 | 1048 |
| 168 | 110 | 69 | 2714 | 2836 |
| 169 | 29 | 29 | 280 | 298 |
| 170 | 20 | 24 | 220 | 234 |
| 171 | 91 | 76 | 3340 | 3258 |
| 172 | 55 | 51 | 1564 | 1476 |
| 173 | 20 | 18 | 232 | 208 |
| 174 | 105 | 88 | 2230 | 2100 |
| 175 | 116 | 95 | 2712 | 2276 |
| 176 | 19 | 17 | 744 | 622 |
| 177 | 37 | 29 | 440 | 346 |
| 178 | 76 | 63 | 1582 | 1244 |
| 179 | 109 | 68 | 1916 | 1618 |
| 180 | 71 | 54 | 1886 | 1884 |
| 181 | 27 | 32 | 800 | 750 |
| 182 | 26 | 17 | 474 | 422 |
| 183 | 46 | 36 | 1178 | 1104 |
| 184 | 48 | 51 | 1130 | 1050 |
| 185 | 94 | 59 | 1394 | 1260 |
| 186 | 85 | 70 | 2490 | 2342 |
| 187 | 43 | 32 | 330 | 232 |
| 188 | 108 | 83 | 3122 | 3092 |
| 189 | 108 | 75 | 2424 | 2308 |
| 190 | 31 | 24 | 716 | 652 |
| 191 | 56 | 41 | 1204 | 1046 |
| 192 | 94 | 79 | 5202 | 4876 |
| 193 | 52 | 33 | 1540 | 1284 |
| 194 | 69 | 61 | 1392 | 1194 |
| 195 | 9 | 4 | 36 | 14 |
| 196 | 131 | 115 | 2562 | 2412 |
| 197 | 61 | 40 | 954 | 976 |
| 198 | 10 | 10 | 62 | 52 |
| 199 | 118 | 88 | 2788 | 3092 |
| 200 | 78 | 58 | 1290 | 1230 |
